# Supplementary material for: Machine-learning-powered extraction of molecular diffusivity from single-molecule images for super-resolution mapping
Source: Commun Biol. 2023 Mar 28;6:336. doi: 10.1038/s42003-023-04729-x (PMC10050076; doi:10.1038/s42003-023-04729-x)
Supplement: Supplementary file 2 — Supplementary Materials [file 42003_2023_4729_MOESM2_ESM.pdf]

Supplementary materials for

Machine-learning-powered extraction of molecular diffusivity from single-molecule images for super-resolution mapping

Ha H. Park[1], Bowen Wang[1], Suhong Moon[2], Tyler Jepson[3], and Ke Xu[1,3*]

Affiliations:

[1]*Department of Chemistry, University of California, Berkeley, CA 94720*

[2]*Department of Electrical Engineering and Computer Sciences, University of California, Berkeley, CA 94720*

[3]*QB3-Berkeley, University of California, Berkeley, CA 94720*

*Correspondence to: xuk@berkeley.edu

Table of contents:

Supplementary figure 1. Architecture of the Pix2D convolutional neural network model.

Supplementary figure 2. Performance assessment with different input channel sizes.

Supplementary figure 3. Evaluation of the data augmentation method for the model training.

Supplementary figure 4. Interpolation and extrapolation performances of the trained model.

Supplementary figure 5. SLB dwell time of BDP-TMR-alkyne single molecules.

Supplementary figure 6. MSD analysis of BDP-TMR-alkyne single molecules diffusing in different SLBs.

Supplementary figure 7. Melting transition of DPPC domains.

Supplementary figure 8. Time-resolved Pix2D results.

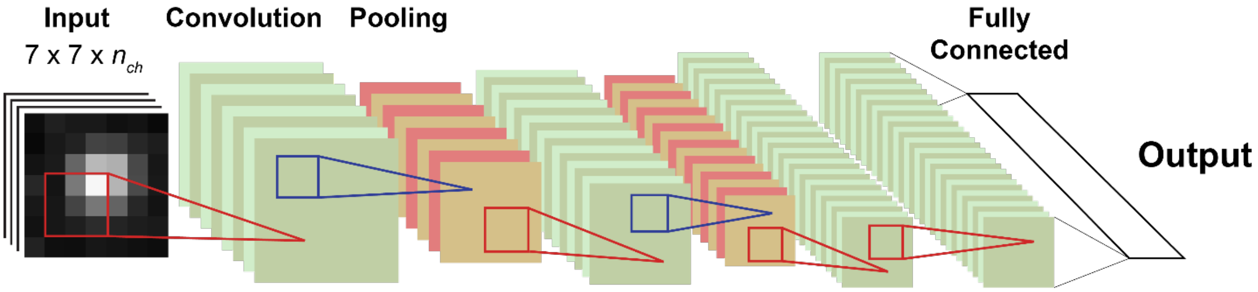

Supplementary figure 1. Architecture of the Pix2D convolutional neural network model. The CNN model processes input through a 3×3 convolution filters of twice the input channel size ($2n_{ch}$), a 2×2 pooling layer applied at stride 2, another 3×3 convolution filters with $4n_{ch}$ channels, another 2×2 pooling layer applied at stride 2, two consecutive 3×3 convolution filters with $8n_{ch}$ channels, followed by a fully connected layer and then final regression layer to the output. The final prediction was computed by a fully connected layer, followed by a regression layer that calculated the MSE losses between the predicted values and the ground truth labels.

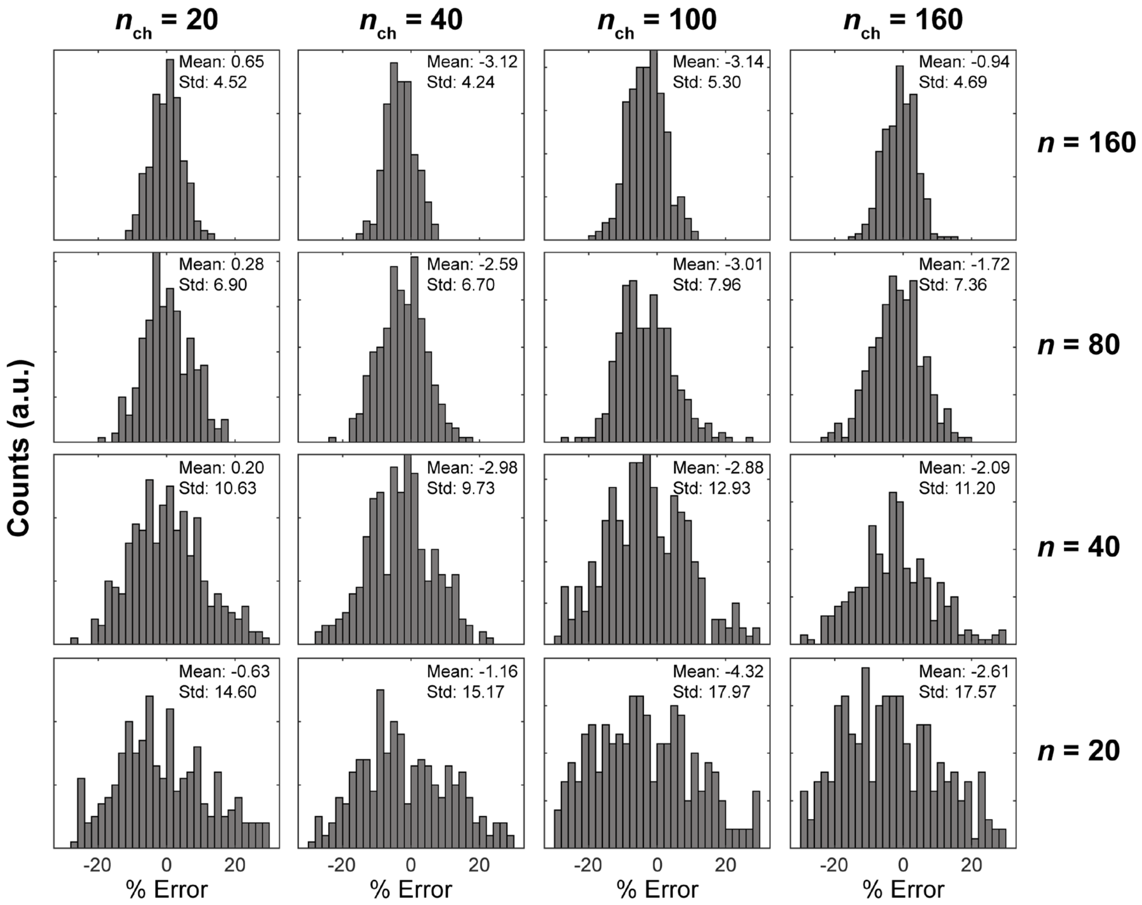

Supplementary figure 2. Performance assessment with different input channel sizes. *n* (equivalent to p^i in each spatial bin when mapping) = 20, 40, 80, and 160 simulated single-molecule images of $D = 2$ μm^2/s were processed with Pix2D of different input channel sizes n_{ch} = 20, 40, 100, and 160. For each dataset, $m = 100$ sets of n_{ch} sampling from *n* images were separately fed into the model as inputs, and the averaged output of the *m* sets was taken as the estimated *D* value. This process was repeated 300 times for each condition by randomly sampling *n* images, and the resultant 300 estimated *D* values were shown as histograms of % error from 2 μm^2/s. a.u., arbitrary unit.

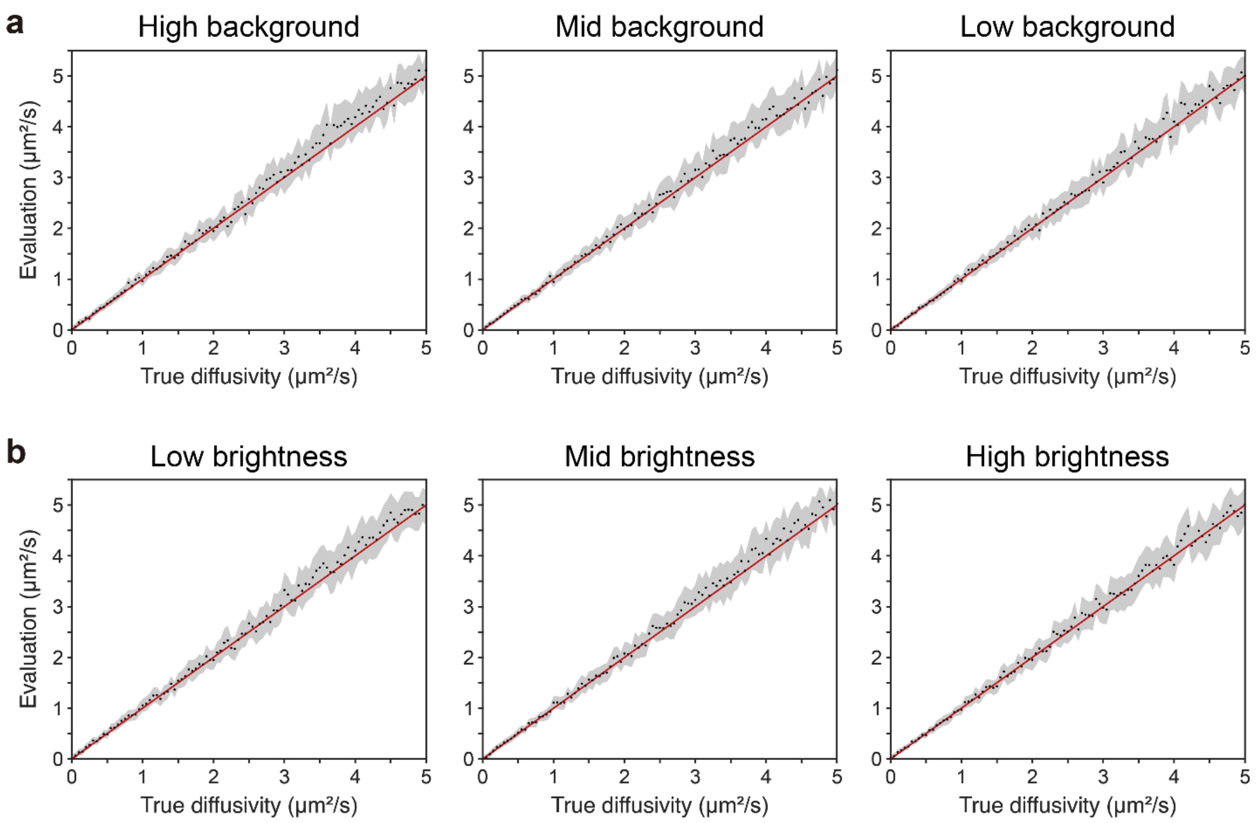

Supplementary figure 3. Evaluation of the data augmentation method for the model training. The model was trained with the data augmentation method of diversifying background noise levels and single-molecule brightness levels. (a) Validations of the model for different simulated background noise levels typical to our experiment data, with input channel size n_{ch} = 40. For each D value, evaluations were performed for 100 sets of n_{ch} images, and the average value and standard deviation were plotted as a black marker and the shaded area, respectively. Red line: reference (evaluation equaling the ground truth). (b) Validations of the model for different simulated single-molecule brightness levels typical to our experimental data.

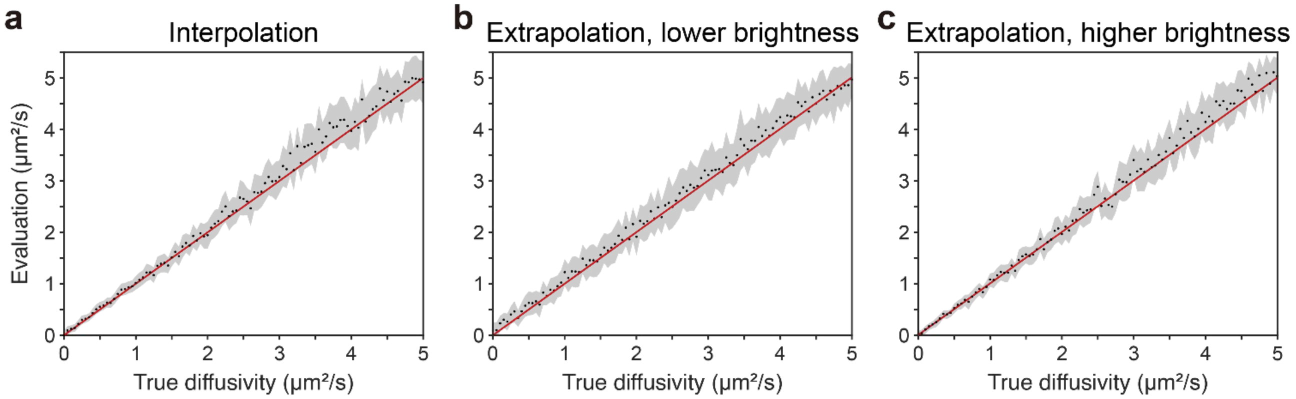

Supplementary figure 4. Interpolation and extrapolation performances of the trained model. Evaluation of the model with simulated datasets with background noise and brightness levels not used for the training, including (a) interpolation with values between the training noise and brightness levels and (b) extrapolation with lower (360 photons) and (c) higher (1350 photons) brightness levels outside the training range.

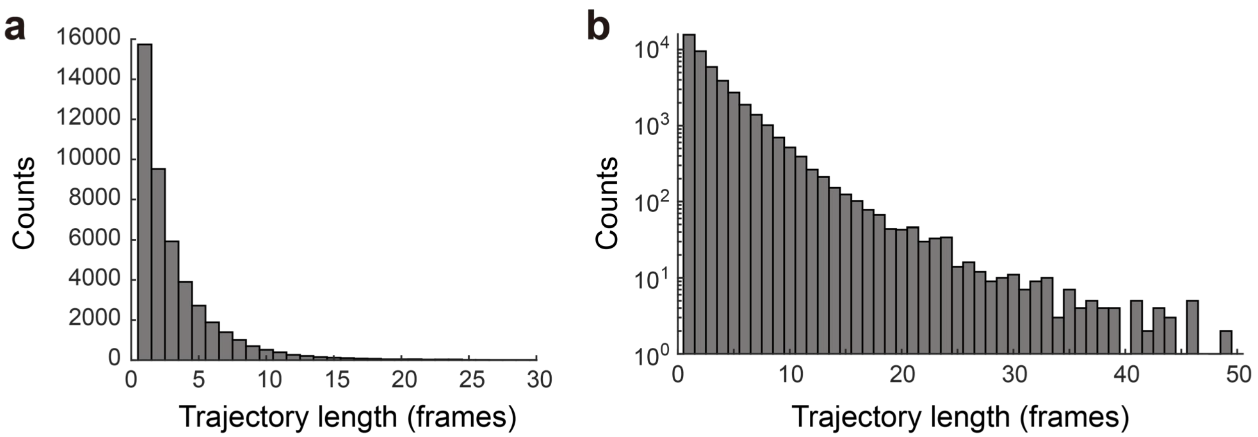

Supplementary figure 5. SLB dwell time of BDP-TMR-alkyne single molecules. Plotted: Distribution of trajectory lengths in a typical experiment for BDP-TMR-alkyne single molecules diffusing in a DOPC SLB, plotted on the linear (a) and logarithmic (b) scales for the counts, respectively. Data was collected at a framerate of 110 frames per second. Tracking was performed using TrackMate[1].

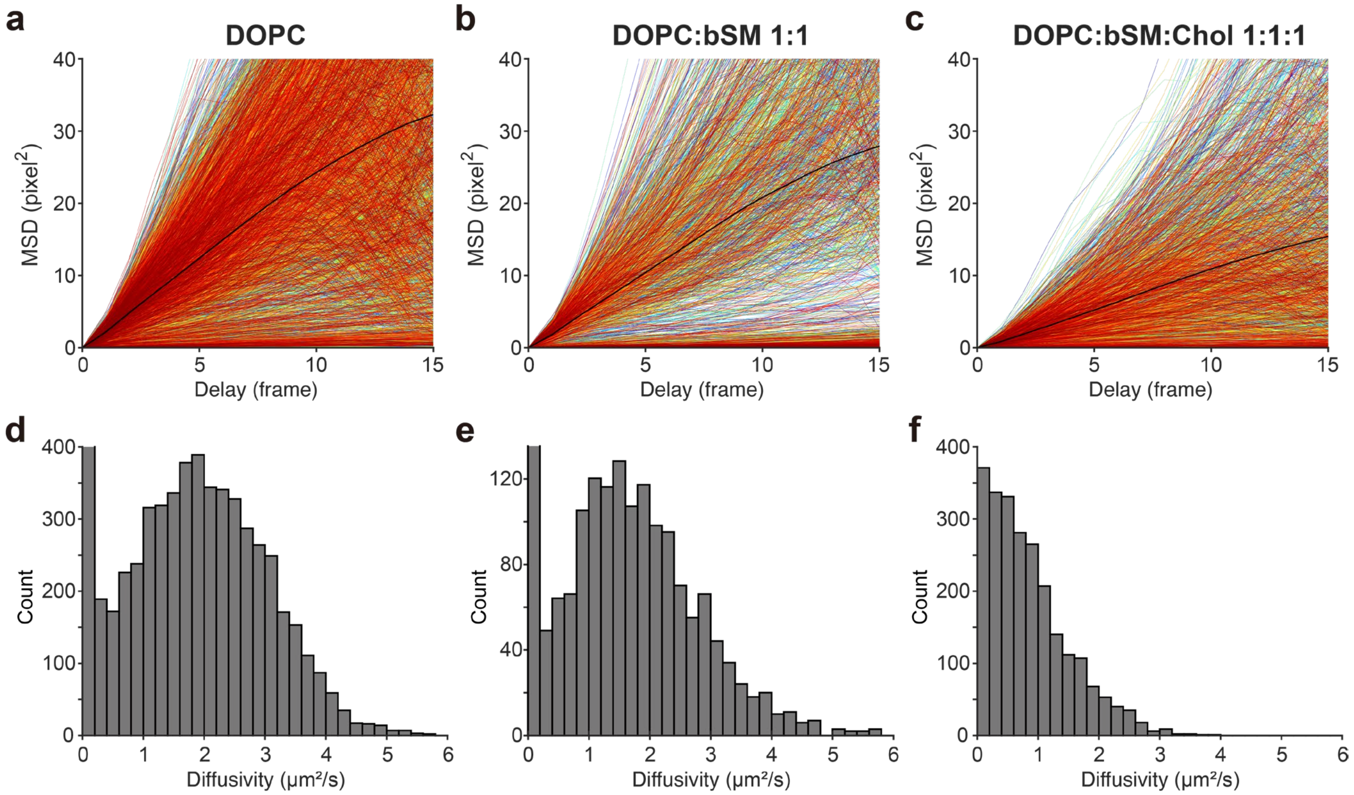

Supplementary figure 6. MSD analysis of BDP-TMR-alkyne single molecules diffusing in different SLBs. MSD analysis was performed using MSDanalyzer[2] for BDP-TMR-alkyne single molecules in the data of Figure 3 for three SLBs compositions: DOPC, DOPC:bSM 1:1, and DOPC:bSM:Chol 1:1:1. Images were acquired at 110 frames per second with a pixel size of 160 nm. Trajectories 15-40 frames in length were selected for the MSD analysis, and the first 25% of MSD curves were used for fitting *D*. *e.g.*, A delay range of 1 to 5 frames was used for a trajectory length of 21. (a-c) MSD curves and weighted-average MSD curves (black) for trajectories in the DOPC (a), DOPC:bSM 1:1 (b), and DOPC:bSM:Chol 1:1:1 (c) SLBs, respectively. (d-f) Distribution of fitted single-molecule *D* values from the MSD curves. Consistent decreasing trends in *D* were observed for the 3 SLBs, even though the distributions were board due to the limited trajectory lengths, and the absolute values generally appeared lower, possibly owing to statistical biases in which the slower-diffusing molecules had higher chances to stay longer in the SLBs to yield the rare long tracks.

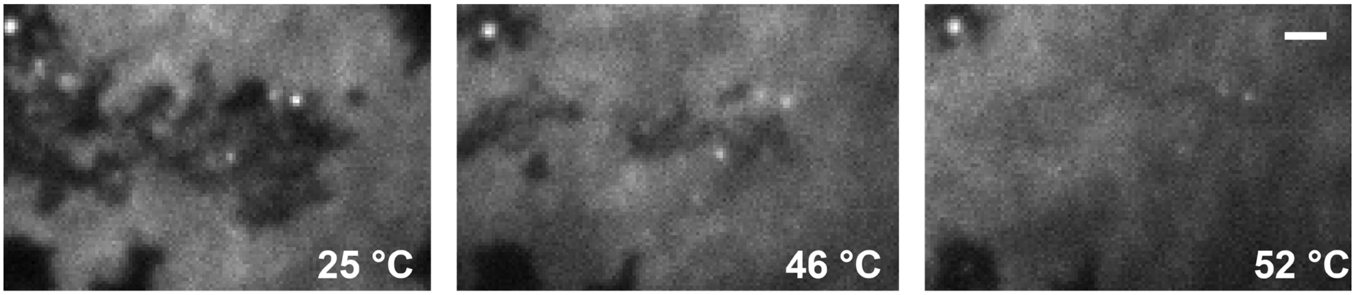

Supplementary figure 7. Melting of DPPC domains. Real-time epifluorescence images of a 60:40 DOPC:DPPC SLB stained with BDP-TMR-alkyne. At room temperature, DPPC domains appeared darker due to limited access for BDP-TMR-alkyne (left). Melting of DPPC domains at elevated temperatures allowed the mixing of DPPC and DOPC domains and BDP-TMR-alkyne intercalation, and so the contrast differences diminished (middle and right). Scale bar: 2 μm.

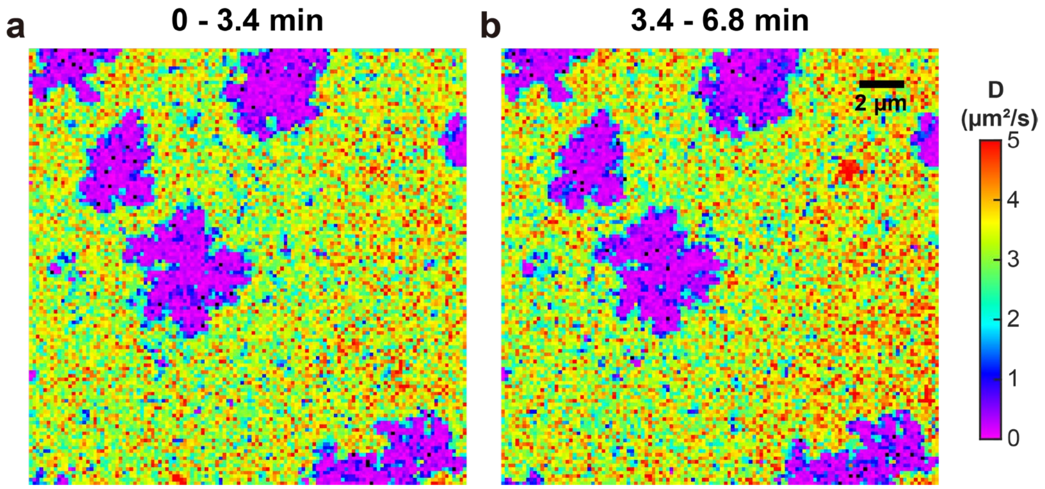

Supplementary figure 8. Time-resolved Pix2D results. The original single-molecule data of Figure 4b collected over 6.8 min was divided into two periods of 3.4-min duration each. The two divided datasets were separately fed into Pix2D to generate two independent diffusivity maps at a 160 nm × 160 nm spatial bin size. *D* maps comparable to Figure 4b were obtained for both periods, thus demonstrating that the diffusion spatial patterns remained unchanged over the recording time, as well as that viable *D* maps can be obtained at ~3 min time resolution.

Reference

1. Tinevez, J.-Y. *et al.* TrackMate: An open and extensible platform for single-particle tracking. *Methods* **115**, 80–90 (2017).
2. Tarantino, N. *et al.* TNF and IL-1 exhibit distinct ubiquitin requirements for inducing NEMO–IKK supramolecular structures. *Journal of Cell Biology* **204**, 231–245 (2014).
